# Euthyroid sick syndrome predicts the risk of ischemic stroke-associated pneumonia in the acute stage of ischemic stroke: a nested case-control study

**DOI:** 10.3389/fendo.2024.1438700

**Published:** 2024-11-11

**Authors:** Shuai Yu, Jia Yan, Robert Logan, Wei-Ting Tang, Jun-Nan Ye, Hong-Xuan Feng, Mei-Xia Wang, Qin-Rong Xu, Xu-Li Jiang, Hai-Yan Lin, Guan-Hui Wu, Qian Gui, Ting-Ting Duan

**Affiliations:** ^1^ Department of Neurology, The Affiliated Suzhou Hospital of Nanjing Medical University, Suzhou Municipal Hospital, Suzhou, Jiangsu, China; ^2^ Biology and Biotechnology Program, School of Science and Technology, Endicott College, Beverly, MA, United States; ^3^ Department of Neurology, The Affiliated Changshu Hospital of Nantong University, Changshu, Jiangsu, China; ^4^ Emergency Medicine Department, The Quzhou Affiliated Hospital of Wenzhou Medical University, Quzhou People’s Hospital, Quzhou, Zhejiang, China; ^5^ Department of Neurology, The Quzhou Affiliated Hospital of Wenzhou Medical University, Quzhou People’s Hospital, Quzhou, Zhejiang, China

**Keywords:** euthyroid sick syndrome, ischemic stroke-associated pneumonia, nested case-control study, ischemic stroke, thyroid hormone

## Abstract

**Objective:**

Ischemic stroke-associated pneumonia (iSAP) affects about 10% of acute ischemic stroke patients during hospitalization. Current prediction scales for iSAP are insufficient. Identifying early biomarkers for stroke-associated pneumonia is crucial for improving patient outcomes. This study aimed to investigate the predictive value of euthyroid sick syndrome (ESS) for iSAP in acute-stage of ischemic stroke patients.

**Methods:**

We studied 1767 acute ischemic stroke patients within one week of symptom onset, categorizing them into an infection group (iSAP, n=376) and control group (control, n=1391). COX regression analysis was used to identify the potential risk and protected factors. Kaplan-Meier time-event curves and Log-Rank tests were performed to differentiate infection time in patients with ESS or normal T3 group.

**Results:**

The iSAP group had higher rates of risk factors like older age, atrial fibrillation, COPD, and ESS, along with elevated levels of WBC, CRP,and FT4 levels (all P < 0.001). Conversely, iSAP patients had lower GCS scores, eGFR, TSH, T3, FT3 (all P < 0.001) and T4 levels (P = 0.005) upon admission. No significant differences were observed in sex, smoking history, hypertension, diabetes, or LDL-C levels (P > 0.05). COX regression analysis identified age, KWST scores, leukocyte count, CRP, and ESS (all P < 0.001) as significantly correlated with iSAP. ROC analysis revealed ESS as a predictor with sensitivity of 35.64% and specificity of 87.92% for SAP prediction, like atrial fibrillation and higher than COPD and eGFR.

**Conclusion:**

ESS at admission predicts a higher risk of stroke-associated pneumonia in acute-stage of ischemic stroke.

## 1 Introduction

Stroke is the second leading cause of death globally and is the third leading cause of disability (1–3). Approximately 60-80% of deaths due to stroke are attributed to ischemic stroke, rather than hemorrhagic or transient stroke (4, 5). Stroke-associated pneumonia (SAP) is a significant early complication following a stroke. During the acute stage of hospitalization for ischemic stroke, about 10% of patients will develop ischemic stroke-related pneumonia (iSAP) (6). SAP is linked to unfavorable functional outcomes and high mortality rates (7, 8). Therefore, early prediction and intervention for stroke-related pneumonia are of paramount importance for improving the prognosis and survival rates of stroke patients. The sensitivity and specificity of commonly used scales for predicting iSAP, such as the A2DS2, AIS-APS, and ISAN, need enhancement through the incorporation of serum biomarkers (9–11). Therefore, identifying biomarkers for early iSAP diagnosis and subsequent treatment initiation is critical for the improvement of ischemic stroke patient prognosis.

Euthyroid sick syndrome (ESS) is one of the prevalent neuroendocrine changes observed in severe illnesses. ESS refers to alterations in thyroid function associated with severe diseases, surgery, and other stressful conditions, primarily characterized by decreased levels of triiodothyronine (T3) and free triiodothyronine (fT3), often without significant changes in thyroid-stimulating hormone (TSH) levels (12). ESS can serve as a predictor of poor outcomes in severe diseases, such as stroke (13–17). However, the relationship between stroke complications, like iSAP, and ESS has not been explored.

Poststroke immunosuppressive syndrome is a contributing factor of poststroke infection (18). Additionally, thyroid hormones influence immune system regulation. Therefore, the predictive value of thyroid hormone level change in poststroke infection is promising. The example of hypothyroidism causing Hashimoto encephalopathy bolsters the hypothesis that thyroid dysregulation could have neurological prognostic value (19–22). Furthermore, the screening of thyroid function upon admission has become a routine diagnostic and treatment measure in most medical institutions in China, setting the stage for prospective studies on the predictive value of thyroid hormones in post-stroke infection (23, 24). This current study aims to analyze the relationship between triiodothyronine level at admission and the incidence of SAP in the acute stages of ischemic stroke. Additionally, it aims to provide insights for accurately predicting iSAP occurrence based on thyroid hormone level changes.

## 2 Methods

### 2.1 Subjects

The subject data for this study were obtained from our previously established observational cohort study on cerebral infarction prognosis. The data were collected from patients in our hospitals (Suzhou Municipal Hospital, Quzhou People’s Hospital and Affiliated Changshu Hospital of Nantong University) who met the inclusion criteria between September 2016 and September 2022. The internally collected data is not yet publicly available. Inclusion criteria required that patients had an acute stroke within one week of enrollment into the study and data collection, were at least 18 years old, had thyroid function tests completed at point of admission, and no exclusion criteria were met. Exclusion criteria included patients with a history of chronic thyroid disease, a history of taking medications that affect thyroxine level or function, non-acute stages of stroke, presence or history of brain tumors, encephalitis, brain trauma, and severe multiple organ dysfunction syndrome. The patient data were categorized into an iSAP group (376 patients), a well-matched control group without iSAP (1391 patients), an ESS group (302 patients), and a well-matched control group with normal T3 levels (1465 patients). This study received approval from the Ethics Committee of all participated Hospital. Data collection did not require the access or use of patient private information. Furthermore, all patients provided informed consent for medical services and clinical study enrollment upon admission.

### 2.2 Research ethics and patient consent

The studies involving human participants were reviewed and approved by ethics committee of the Affiliated Suzhou Hospital of Nanjing Medical University (Suzhou Municipal Hospital), Quzhou Affiliated Hospital of Wenzhou Medical University and Affiliated Changshu Hospital of Nantong University. The patients provided their written informed consent to participate in this study.

### 2.3 Data collection

Demographic and clinical data at admission were collected, including age, sex, smoking history, history of hypertension, type 2 diabetes, atrial fibrillation, and chronic obstructive pulmonary disease (COPD). Baseline clinical characteristics, including stroke severity according to the National Institutes of Health Stroke Scale (NIHSS), dysphagia according to the Kubota water drinking test (KWDT), and disturbance of consciousness according to the Glasgow Coma Scale (GCS). The anatomical structures involved in the stroke and the extent they were involved were determined by brain magnetic resonance imaging (MRI) or cerebral artery computerized tomography (CT) angiography, cervical cerebrovascular color ultrasound, electrocardiogram, and other patient-specific required examination results within 72 hours of admission (25). A NIHSS score of 8 or above is the criteria for severe stroke (26). In addition, we also calculated the Age, Atrial Fibrillation, Dysphagia, Sex, and Stroke Severity (A_2_DS_2_) score, which ranges from 0 to 10 points and is validated for predicting SAP risk (27).

### 2.4 Laboratory tests

Fasting venous blood was evaluated within 24 hours of admission for laboratory tests, including white blood cells, creatinine, low-density lipoprotein cholesterol (LDL-C), homocysteine, CRP and thyroid hormones. The levels of total triiodothyronine (T3), free triiodothyronine (fT3), total thyroxine (T4), free thyroxine (fT4) and thyroid stimulating hormone (TSH) were determined on an ARCHITECT i2000 (Abbott Diagnostics, US) by chemiluminescent microparticle immunoassay methodology (28). ESS was diagnosed with T3 < 1.21 nmol/L or fT3 < 2.63 pmol/L accompanied by normal TSH level (29).

### 2.5 Diagnostic criteria for stroke associated pneumonia

Patients suspected of comorbidity with SAP were diagnosed by two attending neurologists independently according to the Pneumonia In Stroke Consensus group recommendations. The attending respiratory physician was consulted to further establish the diagnosis of SAP, if needed. The diagnosis was made during the first 7 days after the onset of stroke, according to the modified Centers for Disease Control and Prevention (CDC) criteria in patients not receiving mechanical ventilation.

The diagnostic criteria included the presence of at least one of the following indicators: 1. Fever (>38°C) without any other known cause; 2. Abnormal white blood cell count, either leukopenia (<4×10^9^ WBC/L) or leukocytosis (>12×10^9^ WBC/L); 3. Altered mental status with no other identified cause, for adults aged ≥70. Additionally, at least two of the following should be observed: 1. Onset of purulent sputum or a change in sputum character over 24 hours, or an increase in respiratory secretions, or elevated suctioning requirements; 2. Emergence of a new or aggravated cough, dyspnea, or tachypnea (respiratory rate >25/min); 3. Detection of rales, crackles, or bronchial breath sounds; 4. Deterioration in gas exchange, such as O2 desaturation (e.g., PaO2/FiO2 ≤240) or elevated oxygen needs. Furthermore, two sequential chest radiographs should reveal at least one of the following: new or progressive, persistent infiltrate, consolidation, or cavitation. In patients without pre-existing pulmonary or cardiac issues, a single definitive chest radiograph suffices. These criteria were chosen for their relevance to the study’s target population and their established effectiveness in diagnosing pneumonia in stroke patients (30). While these criteria apply to all instances of stroke-associated pneumonia, in our research on ischemic stroke-associated pneumonia, we utilized chest CT, which offers superior clarity compared to the recommended chest X-ray.

### 2.6 Statistical analyses

In our analysis, we employed a range of statistical tests and software tools. Data following a normal distribution were subjected to a t-test, and results are presented as mean ± standard deviation (x-bar ± SD). For data not conforming to a normal distribution, we employed the Mann-Whitney U test, and results are expressed as the median with the lower and upper quartiles. Categorical data were subjected to univariate analysis using the Chi-square test, and the results are reported as the number of cases (percentage). To identify independent risk factors for iSAP, both univariate and multivariate COX regression analyses were conducted. All statistical analyses were carried out using SPSS version 25.0. Additionally, Kaplan-Meier survival curves were generated using GraphPad Prism 9, and group differences in infection-free time were assessed using the Log Rank test. Receiver Operating Characteristic (ROC) curves were created using Medcalc software to evaluate various factors’ predictive performance, including the Area Under the Curve (AUC), sensitivity, and specificity. Statistical significance was defined as a P-value less than 0.05 (α = 0.05).

## 3 Results

In this study, a total of 1,767 patients diagnosed with ischemic stroke were included, with an average age of 69.27 ± 12.46 years. Among the participants, 612 individuals (34.63%) were under 65 years of age, 766 individuals (43.35%) fell into the 65 to 79 age range, while 389 individuals (22.02%) were aged 80 or older. Of the total cohort, 716 were female (40.52%). Regarding thyroid hormone levels, 234 patients (13.24%) exhibited T3 levels below 1.21 nmol/l, while 68 patients (3.85%) had fT3 levels below 2.63 pmol/l. Both T3 and fT3 values fell below the normal range in 57 patients (3.28%). The severity of stroke, as assessed by the NIHSS score, revealed 298 patients (16.86%) with scores ‗ 8 points upon admission. Among them, 149 cases (39.63%) were in the iSAP group, while 149 cases (10.71%) were categorized under the Control group. This difference in the proportion of severe cases between the two groups was statistically significant (χ² = 176.518, P < 0.001). There were 60 cases (19.86%) in the ESS group and 238 cases (16.24%) in the Normal group. There was no significant difference in the proportion of severe cases between these two groups (χ^2^ = 2.343, P = 0.126). Additionally, atrial fibrillation was detected in 292 patients, with 132 cases (35.11%) in the iSAP group and 160 cases (11.50%) in the Control group. The prevalence of atrial fibrillation in the iSAP group was notably higher than that in the Control group (χ² = 119.548, P < 0.001). Similarly, 85 cases of atrial fibrillation (28.15%) were observed in the ESS group, and 207 cases (14.13%) were observed in the Normal group, exhibiting a significantly higher prevalence in the ESS group (χ² = 35.658, P < 0.001).

### 3.1 Baseline characteristics and outcomes of patients with and without iSAP

In our study, we observed notable differences between the iSAP group and the Control group. For example, the proportions of patients in the iSAP group with advanced age, atrial fibrillation, COPD, hyperhomocysteinemia, ESS, high NIHSS score, high KWDT score, high A2DS2 score, elevated white blood cell count, increased CRP levels, and higher fT4 levels were all significantly higher compared to the Control group (all P < 0.001). Conversely, several parameters showed significant differences in the opposite direction. Specifically, the GCS score, eGFR, TSH, T3, fT3 levels (all P < 0.001), and T4 level (P = 0.005) were significantly lower in the iSAP group when compared to the Control group. Notably, there were no significant differences between the two groups in terms of gender, current smoking status, hypertension, diabetes, and LDL-C levels (all P > 0.05). Furthermore, our analysis revealed that 134 cases (35.60%) in the iSAP group exhibited ESS, while only 168 cases (12.10%) in the Control group had ESS. The incongruity in the incidence of ESS between the two groups was highly significant (χ² = 115.953, P < 0.001). Moreover, when examining thyroid hormone levels, we found that T3, T4, fT3, and TSH levels in the iSAP group were significantly lower compared to the Control group (P ≤ 0.005), while the level of fT4 was notably higher in the iSAP group (P < 0.001). These results are presented in **Table 1**.

**Table 1:** Baseline characteristics of patients with iSAP and non-iSAP.

Characteristic	Control (n = 1391)	iSAP (n = 376)	P-value
Age, years, mean ± SD	67.86 ± 12.27	74.49 ± 11.74	< 0.001
Male, n, (%)	840 (60.39)	211 (56.12)	0.134
Current smoking, n (%)	511 (36.74)	138 (36.70)	0.990
Hypertension, n (%)	1071 (76.99)	287 (76.33)	0.786
Diabetes mellitus, n (%)	497 (35.73)	131 (34.84)	0.749
Atrial fibrillation, n (%)	160 (11.50)	132 (35.11)	< 0.001
COPD, n, (%)	56 (4.03)	59 (15.69)	< 0.001
LDL-C, mmol/L, mean ± SD	2.87 ± 0.99	2.80 ± 1.04	0.214
Homocysteine, mmol/L, median (IQR)	14.60 (11.50, 19.00)	16.30 (12.91, 20.08)	< 0.001
eGFR, ml/min/1.73 m^2^, mean ± SD	102.39 ± 33.11	94.83 ± 37.93	< 0.001
NIHSS, median (IQR)	2.00 (1.00, 5.00)	5.00 (2.00, 13.00)	< 0.001
NIHSS > 8 score, n (%)	149 (10.71)	149 (39.63)	< 0.001
GCS, median (IQR)	15.00 (15.00, 15.00)	14.00 (12.00, 15.00)	< 0.001
KWDT, median (IQR)	1.00 (1.00, 1.00)	1.00 (1.00, 4.00)	< 0.001
A_2_DS_2_, median (IQR)	2.00 (2.00, 4.00)	4.00 (3.00, 7.00)	< 0.001
WBC, ×10^9^/L, mean ± SD	6.84 ± 2.09	9.88 ± 3.72	< 0.001
CRP, mg/L, median (IQR)	2.00 (1.00, 4.26)	13.63 (4.00, 37.89)	< 0.001
TSH, mIU/L, median (IQR)	1.72 (1.06, 2.70)	1.33 (0.72, 2.42)	< 0.001
T3, nmol/L, mean ± SD	1.41 ± 0.28	1.23 ± 0.33	< 0.001
T4, nmol/L, mean ± SD	89.81 ± 17.13	86.93 ± 18.71	0.005
FT3, pmol/L, median (IQR)	4.09 (3.55, 4.70)	3.44 (2.91, 4.08)	< 0.001
FT4, pmol/L, median (IQR)	12.56 (10.81, 14.27)	13.50 (11.92, 15.04)	< 0.001
ESS, n (%)	168 (12.08)	134 (35.64)	< 0.001
Body temperature, median (IQR)	36.5 (36.20, 36.70)	36.5 (36.20, 36.80)	0.142

SAP, stroke-associated pneumonia; SD, standard deviation; COPD, chronic obstructive pulmonary disease; LDL-C, low-density lipoprotein cholesterol; IQR, interquartile range; eGFR, glomerular filtration rate; NIHSS, National Institutes of Health Stroke Scale; GCS, Glasgow Coma Scale; KWDT, Kubota water drinking test; A_2_DS_2_, Age, Atrial fibrillation, Dysphagia, Sex, and Stroke Severity; WBC, white blood cell; CRP, C-reactive protein; TSH, thyroid stimulating hormone; T3, triiodothyronine; T4, thyroxine; FT3, free triiodothyronine; FT4, free thyroxine; ESS, euthyroid sick syndrome.

### 3.2 Comparison of infection biomarkers for patients between the ESS and Normal groups

In the ESS group, 134 patients (44.37%) were diagnosed with iSAP, while in the Normal group, 242 patients (16.5%) had iSAP. This difference in iSAP incidence between the two groups was statistically significant (χ² = 115.953, P < 0.001). Additionally, we observed distinct differences in key health indicators. The white blood cell counts and CRP levels were notably higher in the ESS group compared to the Normal group (all P < 0.001). Furthermore, a significantly greater proportion of patients in the ESS group exhibited elevated body temperature upon admission compared to the Normal group (P = 0.004). However, there were no significant differences in overall body temperature between the two groups (P > 0.05). For a comprehensive overview of infection indicators between the ESS and Normal groups, please refer to **Table 2**.

**Table 2:** Infection indicators of patients between Normal group and ESS group. .

Indicator	Normal group (n = 1465)	ESS group (n = 302)	P-value
WBC, ×109/L, mean ± SD	7.22 ± 2.50	8.78 ± 3.77	< 0.001
CRP, mg/L, median (IQR)	2.40 (1.00, 5.62)	5.95 (2.00, 22.73)	< 0.001
Body temperature, median (IQR)	36.50 (36.20, 36.80)	36.50 (36.10, 36.70)	0.210

ESS, euthyroid sick syndrome; WBC, white blood cell; SD, standard deviation; CRP, C-reactive protein; IQR, interquartile range.

### 3.3 Analysis of independent risk factors for ischemic stroke-associated pneumonia

**Figure 1:**
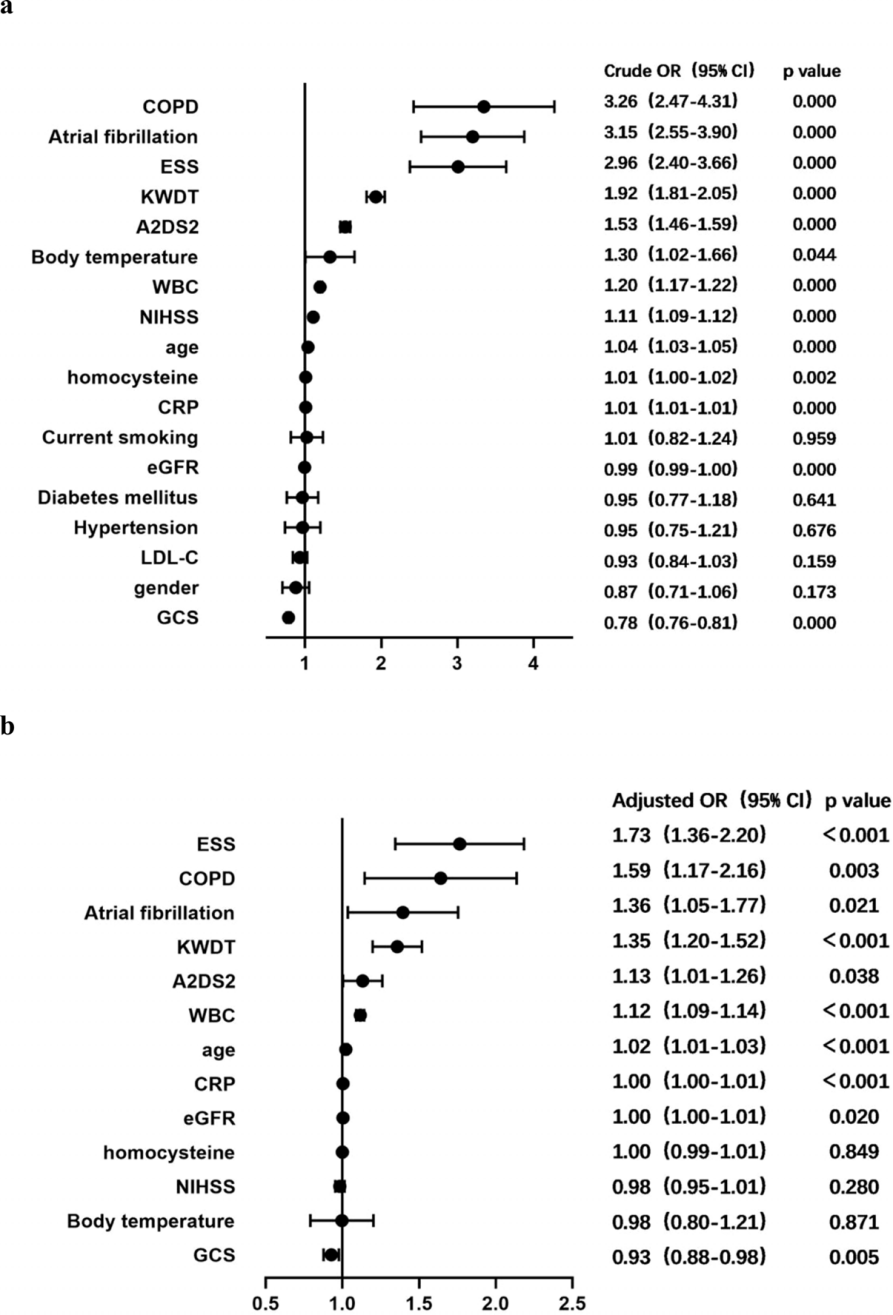
Forest plot of odds ratios for iSAP. **(A)** Crude ORs for potential factors for predicting iSAP. **(B)** Adjusted ORs for potential factors for predicting iSAP. ESS, euthyroid sick syndrome; COPD, chronic obstructive pulmonary disease; CRP, C-reactive protein; KWDT, Kubota water drinking test; eGFR, glomerular filtration rate; NIHSS, National Institutes of Health Stroke Scale; GCS, Glasgow Coma Scale; 95% CI, 95% confidence interval; OR, odds ratio; SAP, stroke-associated pneumonia.

We conducted a rigorous statistical analysis to explore the factors associated with concurrent iSAP using univariate Cox regression analysis. In this analysis, concurrent iSAP served as the dependent variable, while patients’ demographic characteristics, disease-related scores, and hematological indicators were considered as independent variables. The results illuminated several significant relationships. Notably, advanced age, a history of atrial fibrillation or COPD, higher scores on the NIHSS, A2DS2, or KWDT scales, the presence of concurrent ESS, lower eGFR levels, elevated WBC counts, increased CRP levels, higher homocysteine levels, and elevated body temperature were identified as positively associated factors with concurrent iSAP. Conversely, a higher GCS score emerged as a negative associated factor with concurrent iSAP (**Figure 1A**).

Further analysis was conducted by employing concurrent iSAP as the dependent variable, and variables with univariate analysis results showing significance at P < 0.05 were integrated into a multivariate COX proportional risk model. The related factors were thoroughly examined using the enter method at the significance level of α = 0.05. This analysis confirmed that older age, a history of atrial fibrillation or COPD, high scores on the A2DS2 or KWDT scales, the presence of concurrent ESS, lower eGFR levels, and higher levels of WBC counts or CRP levels all independently emerged as positive factors contributing to the occurrence of iSAP. Conversely, a higher GCS score was identified as an independent negative factor for iSAP (**Figure 1B**). These insights into the multifaceted factors associated with concurrent iSAP contributes to our understanding of this complex medical condition.

### 3.4 Kaplan-Meier time-event curves of T3 prediction for infection time in patients with ESS or Normal group

**Figure 2:**
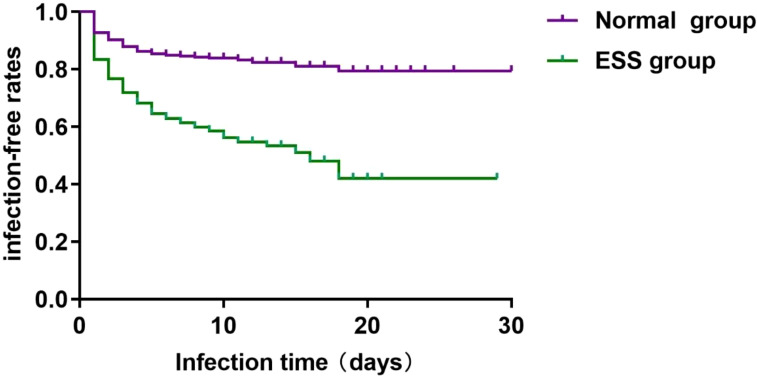
Kaplan-Meier time-event curves and Log-Rank tests for infection time in patients with ESS or Normal group. The median time of infection was 8.0 days (95%CI 3.0-11.0) in ESS group and 9.0 days (95%CI 6.0-11.0) in Normal group (Log Rank P < 0.001).

One month after the admission of ischemic stroke, Kaplan-Meier time event curves of ischemic stroke-associated pneumonia in ESS group and normal group were significantly separated (**Figure 2**). Log-Rank tests for infection time in patients with ESS or Normal group showed the median time of infection was 8.0 days (95%CI 3.0-11.0) in ESS group and 9.0 days (95%CI 6.0-11.0) in Normal group (Log Rank P < 0.001) (**Figure 2**).

### 3.5 The predictive value of various indicators for iSAP

Our analysis of the receiver operating characteristic curve revealed insights into the predictive capabilities of various factors for iSAP. Among these factors, ESS demonstrated a notable area under the curve (AUC) value of 0.618 (95% CI 0.595-0.641), signifying its effectiveness in predicting iSAP. In comparison, COPD (Chronic Obstructive Pulmonary Disease) yielded a lower AUC of 0.558 (95% CI 0.535-0.582, P < 0.05), indicating its comparatively weaker predictive ability for iSAP in this study.

**Figure 3:**
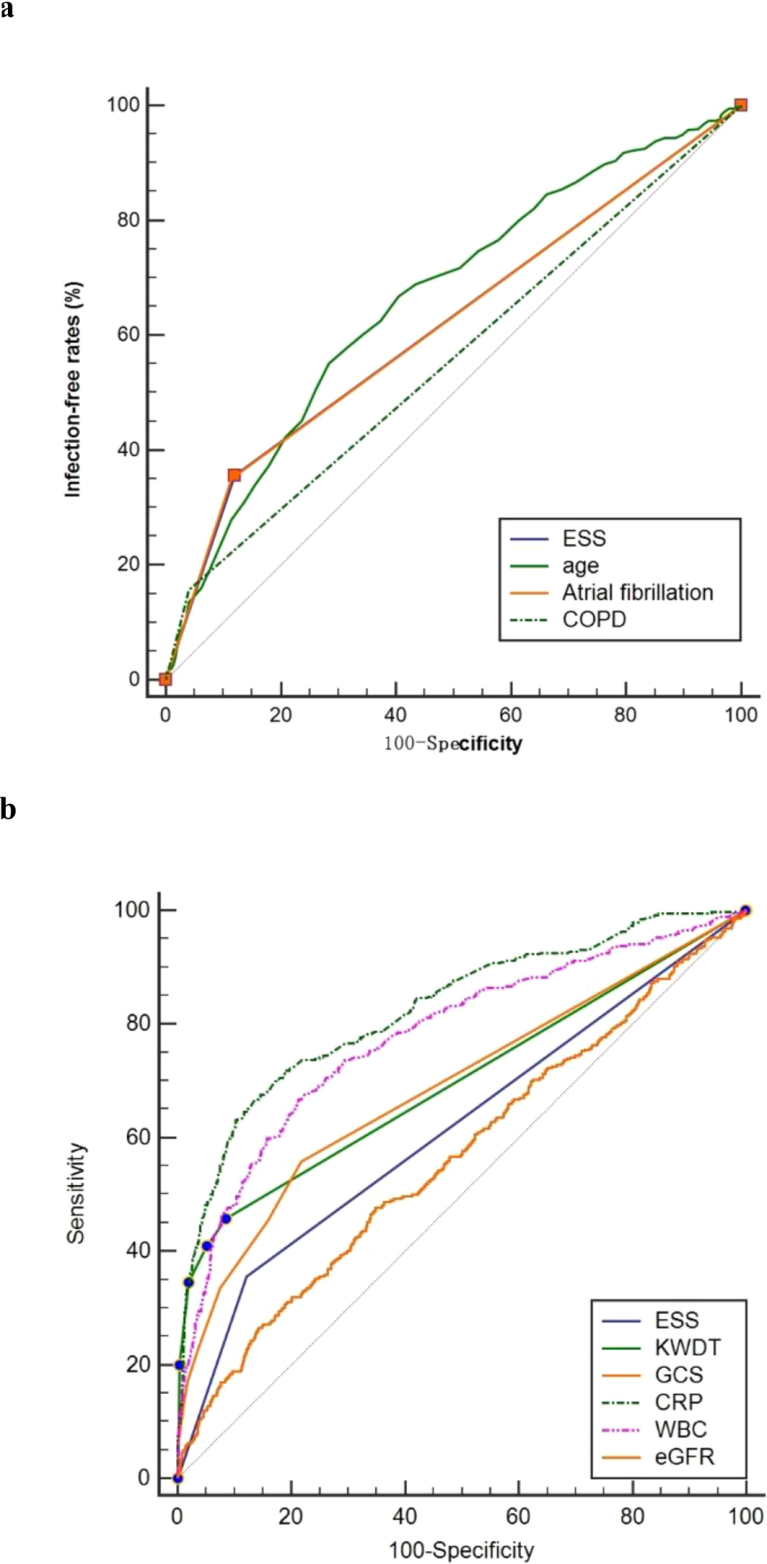
Predictive power comparison between ESS and other factors. ROC curves for ESS, age, Atrial fibrillation, and COPD **(A)**. ROC curves for ESS, KWDT, GCS, CRP, Leukocyte count and eGFR **(B)**. ROC, receiver operating characteristic; AUC, area under the curves.

Furthermore, our analysis highlighted several other factors with their respective AUC values and differential effect compared with ESS: eGFR (AUC = 0.566, 95% CI 0.543-0.590, P=0.0109), CRP (C-Reactive Protein, AUC = 0.826, 95% CI 0.807-0.843, P < 0.0001), WBC (White Blood Cell Count, AUC = 0.778, P < 0.0001), A2DS2 (AUC = 0.758, 95% CI 0.737-0.777, P < 0.0001), KWDT (AUC = 0.698, 95% CI 0.676-0.720, P < 0.0001), GCS (Glasgow Coma Scale, AUC = 0.689, 95% CI 0.667-0.711, P=0.0004), and age (AUC = 0.663, 95% CI 0.641-0.685, P=0.0164). Remarkably, ESS’s predictive power was comparable to that of atrial fibrillation, as both exhibited an AUC of 0.618 (95% CI 0.595-0.641, P=0.9907) (**Figure 3**). These findings underscore the significance of ESS as a predictive factor for iSAP and its competitive performance compared to other variables.

## 4 Discussion

This study underscores ESS as a significant predictor of iSAP during the acute stage of ischemic stroke. It confirms several established risk factors for iSAP, such as older age, atrial fibrillation, COPD, and various inflammatory markers, consistent with prior research (7, 31–34). However, hyperhomocysteinemia and NIHSS scores did not independently predict iSAP. This study also identified that ESS, characterized by reduced T3 levels following ischemic stroke, significantly elevates the risk of iSAP independent of stroke severity. This positions ESS as a vital biomarker for early-stage immune function impairment in severe diseases with infection.

ESS patients with ischemic stroke showed significantly higher WBC counts upon admission than those with normal T3 levels, further confirming ESS’s predictive value for iSAP. T3 plays a pivotal role in immune function, impacting various immune cell activities. For example, T3 promotes proliferation and cytotoxicity in T cells, neutrophils, macrophages, and natural killer cells, and increases the production of pro-inflammatory cytokines such as IL-12, IL-6, and TGF-β (35–37). Lower T3 levels compromise immune function, elevating infection risk.

Elevated CRP levels were observed in ischemic stroke patients with ESS. Both ESS and elevated CRP levels independently predict an increased risk of iSAP, even without elevated body temperature (14, 38). Elevated CRP is widely used to monitor acute stress conditions like cardiovascular or cerebrovascular diseases, infections, and surgical procedures. Given that ESS can co-occur with acute stress or chronic disease states, its correlation with elevated CRP levels is not surprising. The predictive value of body temperature at admission for SAP remains uncertain, especially as some elderly patients with pulmonary infections may not exhibit fever symptoms (38, 39). In our study, body temperature upon admission did not independently predict iSAP, possibly due to the low proportion of iSAP patients with fever. Further research is needed to explore its potential utility in specific patient subgroups.

Other studies have confirmed ESS’s ability to predict infection severity and prognosis (40, 41). While a few single-center retrospective studies have reported ESS’s predictive outcomes for SAP or stroke-related infection, they have neglected the temporal aspects and lacked information on the sensitivity and specificity of the diagnostic data (14, 42). In our study, ESS exhibited a specificity of 87.92%, sensitivity of 35.64%, and an AUC of 0.618 for predicting iSAP. ESS’s predictive performance for iSAP paralleled that of atrial fibrillation and surpassed that of COPD and eGFR. Atrial fibrillation has been adopted as a predictor of SAP by several scales (43, 44). Furthermore, hypothyroidism increases the risk of atrial fibrillation (45–49). Although ESS’s predictive performance has a relatively poor sensitivity and a marginally satisfactory AUC, it exhibits strong specificity and performs well in conjunction with other clinical benchmarks.

The inhalation of oropharyngeal flora due to dysphagia is a primary cause of SAP (50). Patients with severe strokes are at higher risk of oropharyngeal flora colonization in the alveoli and have a reduced cough reflex, promoting the development of SAP (51). The KWDT score is a widely used tool for assessing swallowing function with high sensitivity and specificity (52). In our study, the KWDT score’s correlation with iSAP prediction was stronger than the NIHSS score ability to predict iSAP risk. This is likely due to the relatively smaller proportion of the NIHSS focusing on dysphagia at the expense of including factors like motor function or level of consciousness. The relevance of the KWDT score allows it to independently predict the risk of iSAP in our study.

There are several limitations in this study. First, the ability of nested case-control studies to retrospectively evaluate the level of influencing factors depends on the level of influencing factors of the patients included in the cohort. Differences in exposure factors between cohort studies and nested case-control studies may lead to selection bias and result bias. Second, a single test of T3 level on admission may not fully reflect the changes of T3 concentration in different periods of AIS and its influence on the prognosis. Third, a single factor approach was not effective in predicting iSAP risk in this study, as with previous studies. This study did not explore which combination of risk factors could effectively predict iSAP. Although there are limitations, this study is the first to report the efficacy of ESS in predicting stroke-associated pneumonia in the acute stage of ischemic stroke, and confounding factors were well controlled, which provides clear evidence for the effect of ESS in predicting iSAP.

## Data Availability

The original contributions presented in the study are included in the article/Supplementary Material. Further inquiries can be directed to the corresponding authors.
